# Associations Between Cigarette Smoking and Poor Sleep Among Adults With a Lifetime Cancer Diagnosis

**DOI:** 10.1002/cnr2.70386

**Published:** 2025-11-21

**Authors:** Nazife Pehlivan, Andrea Villanti, Michael B. Steinberg, Ho Kim, Tanya R. Schlam, Chaelin K. Ra

**Affiliations:** ^1^ Graduate School of Public Health Seoul National University Seoul South Korea; ^2^ Rutgers Cancer Institute of New Jersey, Robert Wood Johnson Medical School Rutgers University New Brunswick New Jersey USA; ^3^ Institute for Nicotine and Tobacco Studies Rutgers University New Brunswick New Jersey USA; ^4^ Department of Kinesiology University of Wisconsin‐Madison Madison Wisconsin USA

**Keywords:** cancer, cigarette, sleep, smoking

## Abstract

**Background:**

Poor sleep is associated with cigarette smoking and cancer diagnosis, but little is known about the contribution of smoking to poor sleep following a cancer diagnosis.

**Methods:**

Using the National Health and Nutrition Examination Survey (*N* = 6183), multivariable Poisson regression models estimated the associations between lifetime cancer diagnosis, cigarette smoking, and poor sleep, controlling for covariates and evaluating interactions between smoking and cancer diagnosis.

**Results:**

Among adults, 11.6% reported a lifetime cancer diagnosis, and 13.3% of those reported current cigarette smoking. Adults with a cancer diagnosis who smoked had a higher prevalence of inappropriate sleep duration (Adjusted Prevalence Ratio, APR: 2.29, 95% CI: 1.03, 5.13) and snorting/stop breathing (APR: 1.63, 95% CI: 1.10, 2.41) than those without a cancer diagnosis who don't smoke.

**Conclusion:**

Smoking among adults with a lifetime cancer diagnosis is correlated with poor sleep, highlighting the need for targeted smoking cessation interventions to improve sleep health in this population.

## 1 Introduction

While many cancer patients who smoke cigarettes are motivated to quit smoking after their cancer diagnosis and make quit attempts, more than half continue to smoke [1, 2]. As a result, the prevalence of cigarette smoking is similar among adult cancer survivors (11.4%) [3] and the general US adult population (11.6%) [4]. However, cancer patients who quit smoking may benefit from improved all‐cause mortality, quality of life, and overall health [5].

Several studies have documented the association between cigarette smoking and poor sleep [6, 7, 8]. Symptoms of poor sleep include a decreased sleep duration, sleep latency, nighttime awakenings, and daytime dysfunction [9]. Smoking is known to be associated with both insufficient or excessive sleep duration [7, 10, 11] and other indicators of poor sleep such as snoring, snorting/breathing cessation, daytime sleepiness [10, 12], and trouble sleeping [10, 12]. Poor sleep is also known to be a problem among cancer patients [9, 13]. Because of the high prevalence of poor sleep among cancer patients, sleep‐related research has been emphasized as one of the research priorities by the Oncology Nursing Society [14]. While poor sleep and smoking are related, the independent and joint effects of smoking and cancer diagnosis on sleep outcomes remain unclear.

This study aims to investigate the associations between current smoking status and poor sleep outcomes (e.g., inadequate sleep duration, snoring, snorting, daytime sleepiness, and sleep disturbances) in adults with a lifetime cancer diagnosis compared to adults without cancer. We hypothesize that smoking is positively associated with poor sleep, and that this association is stronger among adults with a lifetime cancer diagnosis than among those without cancer.

## 2 Methods

### 2.1 Data

National Health and Nutrition Examination Survey (NHANES data 2017–March 2020; pre‐pandemic) data was used to examine how the associations between current smoking status and sleep outcomes differ by cancer diagnosis among American adults. The final sample was 6183 individuals aged 20 or over. The analyses were weighted to account for the complex sample design. More information about the sample design, sampling weights, and survey are available in NHANES guidelines [15]. The study procedure was reviewed by the Institutional Review Board at Seoul National University (IRB No.: E2303/003–007).

### 2.2 Measures

The main predictors of sleep outcomes were current smoking status and lifetime cancer diagnosis. Lifetime cancer diagnosis was a binary variable assessed by the survey question “Have you ever been told by a doctor or other health professional that you had cancer or a malignancy of any kind?” asked to adults aged 20 and over. Current smokers were those who reported having smoked at least 100 cigarettes during their lifetimes and smoked every day or some days at the time of the interview. We used binary classification of current smoking status (current smoker: 1 vs. non‐smoker: 0). Inappropriate sleep hours were categorized based on the age‐specific appropriate sleep duration from the National Sleep Foundation (sleep fewer or greater than 6–11 h for ages 18–25; 6–10 h for ages 26–64; 5–9 h for ages 65 and older) [16]. Sleep trouble was a self‐reported response to the survey question “Have you ever told a doctor or other health professional that you have trouble sleeping?” Sleep outcomes were dichotomized: sleep duration (not appropriate: 1 vs. likely appropriate: 0), snoring (rarely/occasionally/frequently: 1 vs. never: 0), snorting (rarely/occasionally/frequently: 1 vs. never: 0), daytime sleepiness (rarely/sometimes/often/almost always: 1 vs. never: 0), and sleep trouble (yes: 1 vs. no: 0). Covariates were age group (20–25 vs. 26–64 vs. 65+), sex (female vs. male), marital status (married/living with a partner vs. other), ethnicity (Non‐Hispanic White vs. Non‐Hispanic Black vs. other), body mass index (BMI, 18.5–25 vs. other), heavy drinking (heavy drinker: 4+ drinks/day for females and 5+ drinks/day for males vs. otherwise), depressive symptoms (none‐to‐mild vs. clinically significant), socioeconomic status (SES) was proxied by poverty status (at/above vs. below 200% of the poverty threshold). Obesity is a risk factor for increasing sleep‐disordered breathing [17]. Alcohol drinking, depression, and low SES are known to increase the risk of sleep problems [18].

### 2.3 Analyses

Quasi‐Poisson regression models were used to estimate direct effects in the form of prevalence ratios (PR) for the five sleep outcomes by current cigarette smoking and cancer diagnosis, unadjusted and adjusted for covariates. Then, in separate models, potential interaction between current cigarette smoking and cancer diagnoses was evaluated by including a multiplicative term. All analyses were performed using RStudio (Version 4.2.2) and accounted for survey weights using the Survey package [19].

## 3 Results

Among 6183 respondents 1117 (16.6%) were current smokers and 678 (11.6%) reported a cancer/malignancy diagnosis (Table 1). The mean age of the respondents was overall 48.4 where adults with a lifetime cancer diagnosis were 63.0 years old and adults without cancer were 46.5. Slightly more than half of the sample was female (52.0). Self‐reported ethnicity of the sample was composed of 66.4% non‐Hispanic white, 10.5% non‐Hispanic black, and 23.1% other ethnicities. Among adults with a lifetime cancer diagnosis, 13.3% were current smokers whereas 17.1% were among adults without cancer.

**TABLE 1:** Characteristics of US adults with and without a cancer diagnosis in their lifetime, National Health and Nutrition Examination Survey, 2017–2020.

	All		Lifetime cancer/malignancy diagnosis		No lifetime cancer/malignancy diagnosis		*p*
	*N*	%	*N*	%	*N*	%	
Sex							
Male	3015	48.04	317	40.9	2698	47.04	*0.043*
Female	3168	51.96	361	59.1	2807	52.96	
Marital status							
Married/living with partner	3648	64	387	62.87	3261	64.15	*0.705*
Single	2535	36	291	37.13	2244	35.85	
Ethnicity							
NH White	2385	66.42	415	82.83	1970	64.26	*< 0.001*
NH Black	1576	10.48	121	5.96	1455	11.07	
Other	2222	23.11	142	11.21	2080	24.67	
Body mass index							
Normal	1485	24.85	141	20.68	1344	25.39	*0.129*
Other	4698	75.16	537	79.32	4161	74.61	
Heavy drinker							
No	5549	88.95	650	96.65	4899	87.94	*< 0.001*
Yes	634	11.05	28	3.35	606	12.06	
Depressive symptoms							
None‐to‐mild	5639	91.52	600	89.31	5039	91.81	*0.297*
Clinically significant	544	8.48	78	10.69	466	8.19	
Socioeconomic status (SES)							
At/Above 200% poverty threshold	3093	62.84	407	75.2	2686	61.22	*< 0.001*
Below 200% poverty threshold	3090	37.16	271	24.8	2819	38.79	
Smoking status							
Non‐smoker	5066	83.37	583	86.69	4483	82.93	*0.054*
Current smoker	1117	16.63	95	13.31	1022	17.07	

	M	SD	M	SD	M	SD	
Age	48.44	17.01	63.03	13.71	46.53	16.46	*< 0.001*
*Unweighted N (Total)*	*6183*	*100*	*678*	*11.61*	*5505*	*88.39*	

*Note:* National Health and Nutrition Examination Survey (NHANES) 2017–2020 pre‐pandemic. Prevalence estimates are weighted to reflect survey design and differential non‐response.

Abbreviations: %, weighted percentage; M, mean; *N*, raw sample size; NH, Non‐Hispanic; SD, standard deviation (M and SD were provided only for “Age” under columns *N* and %, respectively).

Table 2 shows the unweighted sample sizes and survey‐weighted prevalence and prevalence ratios of five poor sleep outcomes (i.e., inappropriate sleep duration, snoring, snorting, daytime sleepiness, sleep trouble). Generally, there were few differences in these outcomes by lifetime cancer diagnosis or current smoking status. The prevalence of sleep trouble was higher among the respondents with a lifetime cancer diagnosis compared to those without cancer diagnoses (39.0% vs. 30.8%; PR 1.28) and among those who smoked cigarettes compared with non‐smokers (PR 1.19); however, these relationships were not significant after adjusting for covariates. Current smoking was positively associated with snoring (Adjusted Prevalence Ratio, APR: 1.07, 95% CI: 1.01, 1.13). Interactions indicated that, in comparison to adults without cancer who do not smoke (Reference), those with a lifetime cancer diagnosis and currently smoke cigarettes were more likely to suffer from an inappropriate sleep duration (APR: 2.29 [95% CI: 1.03, 5.13]) and snorting/stop breathing during sleep (APR 1.63 [95% CI: 1.10, 2.41]).

**TABLE 2:** Unweighted sample sizes (*n*) and survey‐weighted prevalence (%) and prevalence ratios (PR) of sleep outcomes.

	Inappropriate sleep duration (*n* = 6183)	Snoring^a^ (*n* = 5780)	Snorting/stop breathing^a^ (*n* = 5862)	Daytime sleepiness^b^ (*n* = 6179)	Sleep trouble (*n* = 6180)
	Yes	Yes	Yes	Yes	Yes
Lifetime cancer/malignancy diagnoses					
Unweighted sample size (*n*)	837	3851	1299	4616	1586
Weighted prevalence (%)	12.05	74.44	24.37	86.94	30.76
No lifetime cancer/malignancy diagnoses					
Unweighted sample size (*n*)	111	447	184	577	263
Weighted prevalence (%)	12.42	72.11	28.07	84.99	38.98
Direct effects prevalence ratios (PR)					
Lifetime cancer/malignancy diagnoses					
No (Reference)	—	—	—	—	—
Yes					
Unadjusted PR	1.05	0.97	1.16	0.98	1.28
	(0.76, 1.44)	(0.87, 1.09)	(0.94, 1.44)	(0.94, 1.02)	(1.07, 1.52)
Adjusted PR^c^	0.95	0.96	1.18	0.97	1.06
	(0.70, 1.28)	(0.85, 1.07)	(0.98, 1.42)	(0.93, 1.03)	(0.87, 1.30)
Current smoking status					
Non‐smoker (Reference)	—	—	—	—	—
Current smoker					
Unadjusted PR	1.42	1.04	1.16	1.02	1.19
	(0.98, 2.04)	(0.98, 1.11)	(0.93, 1.45)	(0.97, 1.07)	(1.07, 1.32)
Adjusted PR^c^	1.26	**1.07**	1.04	1.01	1.08
	(0.88, 1.81)	**(1.01, 1.13)**	(0.81, 1.34)	(0.96, 1.06)	(0.94, 1.24)
Interaction effects prevalence ratios (PR)					
Lifetime cancer/malignancy diagnoses: current smoking status					
No: No (Reference)	—	—	—	—	—
Yes: Yes (Unadjusted PR)	**2.24**	0.94	**1.65**	1.02	1.02
	**(1.04, 4.81)**	(0.83, 1.07)	**(1.17, 2.32)**	(0.90, 1.14)	(0.68, 1.53)
Yes: Yes (Adjusted PR^c^)	**2.29**	0.95	**1.63**	1.02	1.04
	**(1.03, 5.13)**	(0.83, 1.10)	**(1.10, 2.41)**	(0.89, 1.16)	(0.65, 1.66)
Observations	6183	5780	5862	6179	6180

*Note:* Bold values indicate statistically significant associations at *p* < 0.05.

^a^ Yes, rarely/occasionally/frequently; No, never.

^b^ Yes, rarely/sometimes/often/almost always; No, never.

^c^ Multivariable Survey‐Weighted Poisson Regression Models control for sex, age group (20–25 vs. 26–64, 65+), marital status (married living with partner vs. single), ethnicity (Non‐Hispanic white vs. Non‐Hispanic Black, Other), body mass index (normal vs. otherwise), heavy drinker (no vs. yes), depressive symptoms (none‐to‐mild vs. clinically significant), socio‐economic status (at/above 200 poverty threshold vs. below 200 poverty threshold), and the interaction between Current Smoking Status and Lifetime Cancer/Malignancy Diagnoses.

Figure S1 shows the odds of having sleep disturbances for each subgroup by current smoking status and cancer/malignancy diagnoses (i.e., (a) no smoking and no cancer (reference), (b) yes smoking and no cancer, (c) no smoking and yes cancer, and (d) yes smoking and yes cancer). Current smokers with cancer were 1.99 (95% CI: 1.16, 3.05) times more likely to suffer inappropriate sleep duration compared to the reference group. Current smokers with a cancer diagnosis were 1.79 (95% CI: 1.17, 2.43) times more likely to snort/cease breathing during sleep compared to the reference group. Other groups (i.e., (b) yes smoking and no cancer and (c) no smoking and yes cancer) for both outcomes did not show any significant differences compared to the reference group.

## 4 Discussion

Findings from this nationally representative sample support previous findings showing that current smoking was associated with more frequent snoring [20, 21, 22]. In this study, current smoking was associated with increased snoring which may be explained by smoking‐induced irritation and inflammation in the upper airways [21] and the absence of nicotine during sleep hours [20]. Given that we used data from the entire US, our results are generalizable to the civilian, non‐institutionalized adult population in the United States.

Importantly, we found that adults with a lifetime cancer diagnosis who currently smoke were at significantly greater risk of experiencing an inappropriate sleep duration and snorting/stop breathing during sleep compared to individuals without cancer who do not smoke. This interaction highlights a subgroup particularly vulnerable to compounded sleep disturbances—those managing both cancer and ongoing tobacco use. The mechanisms may include smoking‐related respiratory dysfunction, nicotine withdrawal effects, and cancer‐ or treatment‐related vulnerabilities to poor sleep [20, 23].

While several studies investigated the associations between sleep outcomes vs. smoking and sleep outcomes vs. cancer separately, this study is among the first to examine the joint effects of smoking and cancer history on sleep outcomes. Our results suggest that smoking may amplify sleep‐related problems in adults with a cancer diagnosis, underscoring the importance of tailored smoking cessation efforts for this population. Addressing smoking in survivorship care may offer dual benefits—reducing tobacco‐related health risks and improving sleep quality, which is often impaired among cancer survivors. This study has several limitations. Firstly, we used cross‐sectional data to examine the poor sleep outcomes, which did not confirm the temporal order among the variables we used. Secondly, the participants self‐reported sleep outcomes, which may have resulted in recall bias. Finally, we could not divide cancer/malignancy diagnoses into specific types of cancer/malignancy due to the insufficient size of subsamples. Therefore, our results do not inform the impacts of smoking on sleep outcomes among patients with specific cancer/malignancy types.

## 5 Conclusion

Targeting smoking behaviors following a cancer diagnosis may improve not only long‐term health outcomes but also sleep quality, which is frequently disrupted among cancer survivors. Our findings suggest that current smoking may exacerbate sleep disturbances—particularly sleep duration and breathing‐related issues—among individuals with a cancer history. These results underscore the importance of integrating sleep health into clinical care and survivorship planning. Smoking cessation interventions tailored to cancer survivors could have dual clinical benefits: reducing the risk of cancer‐related complications and improving sleep, which in turn may enhance quality of life, treatment adherence, and recovery outcomes.

While our study did not include data on changes in sleep disturbances after smoking cessation among cancer survivors, existing literature on this specific relationship is limited. This gap highlights an important direction for future research to examine whether quitting smoking can directly improve sleep outcomes in this population. Future research should also investigate the temporal associations between smoking and sleep among cancer survivors using longitudinal data, and consider incorporating objective sleep measures through mobile health technologies to reduce recall bias and capture real‐time sleep patterns.

## Author Contributions

**Nazife Pehlivan:** conceptualization (equal), data curation (lead), formal analysis (lead), methodology (equal), visualization (lead), writing – original draft (equal), writing – review and editing (equal). **Andrea Villanti:** supervision (equal), writing – review and editing (equal). **Michael B. Steinberg:** supervision (equal), writing – review and editing (equal). **Ho Kim:** supervision (equal), writing – review and editing (equal). **Tanya R. Schlam:** writing – review and editing (equal). **Chaelin K. Ra:** conceptualization (equal), methodology (equal), supervision (equal), validation (lead), writing – original draft (equal), writing – review and editing (equal).

## Conflicts of Interest

The authors declare no conflicts of interest.

## Supporting information


**Data S1:** Supporting Information.

**Figure S1:** Odds ratios of sleep disturbances by cancer/malignancy diagnoses and smoking status.

## Data Availability

The data that support the findings of this study are openly available in Vital Health Statistics at https://stacks.cdc.gov/view/cdc/115434/cdc_115434_DS1.pdf.
