# Supplementary material for: Associations Between Cigarette Smoking and Poor Sleep Among Adults With a Lifetime Cancer Diagnosis
Source: Cancer Rep (Hoboken). 2025 Nov 21;8(11):e70386. doi: 10.1002/cnr2.70386 (PMC12637855; doi:10.1002/cnr2.70386)
Supplement: Supplementary file 1 — Data S1: Supporting Information. [file CNR2-8-e70386-s001.pdf]

심의 면제 확인서

수신

**연구책임자**	**이름:** 김호	**소속:** 보건대학원 보건학과	**직위:** 교수
**지원기관**	해당없음		

과제정보

<table>
<tr><td colspan="2">승인번호</td><td>IRB No. E2303/003-007</td></tr>
<tr><td colspan="2">연구과제명</td><td>미국 암/악성 병력이 있는 성인 중에서 흡연과 수면의 관련</td></tr>
<tr><td colspan="2">연구종류</td><td>공개된 정보 이용 연구</td></tr>
<tr><td colspan="2">면제일자</td><td>2023-03-20</td></tr>
<tr><td colspan="2">심의결과</td><td>면제승인</td></tr>
<tr><td>검토의견</td><td>면제 검토의견</td><td>본 연구는 공개된 자료인 Centers for Disease Control and Prevention의 National Health and Nutrition Examination Survey Data를 이용하는 연구로서 「생명윤리 및 안전에 관한 법률 시행규칙」 제13조 제1항 제3호에 근거하여 심의를 면제합니다.</td></tr>
</table>

상기 연구과제에 대하여 본 위원회에서는 심의면제대상임을 확인합니다.

2023년 03월 20일

서울대학교 생명윤리위원회 위원장

본 위원회가 승인한 연구를 수행하는 연구자들은 다음의 사항을 준수해야 합니다.

1. 모든 연구자들은 아래의 사항을 준수하여야 합니다.
2. 연구자께서는 제출하신 계획서에 따라 연구를 수행하여야 하며, 이와 다르게 연구를 진행하실 경우 다시 심의를 진행하셔야 함을 유의하시기 바랍니다.
3. 위원회의 요구가 있을 때에는 연구의 진행과 관련된 보고를 위원회에 제출하여야 합니다.
4. 연구윤리를 위하여 관련부처가 필요시 조사 및 감독 차원에서 현장점검을 실시할 수 있습니다.
5. 연구와 관련된 기록은 연구가 종료된 시점을 기준으로 최소 3년간 보관하여야 합니다.
