# Supplementary material for: Associations Between Cigarette Smoking and Poor Sleep Among Adults With a Lifetime Cancer Diagnosis
Source: Cancer Rep (Hoboken). 2025 Nov 21;8(11):e70386. doi: 10.1002/cnr2.70386 (PMC12637855; doi:10.1002/cnr2.70386)
Supplement: Supplementary file 2 — Figure S1: Odds ratios of sleep disturbances by cancer/malignancy diagnoses and smoking status. [file CNR2-8-e70386-s002.docx]

**Supplement – Figure S1**

**Figure S1. Odds Ratios of Sleep Disturbances by Cancer/Malignancy Diagnoses and Smoking Status**

**Notes:**

Reference Group: The subgroup of adults who were never diagnosed with any cancer/malignancy and are not smokers (OR:1.00).

All ORs are adjusted for covariates. 95% confidence intervals for significant ORs are included in the manuscript.

* indicates significant odds ratios at p<0.05.
